# Direct Solids Analysis Using Sputter Initiated Resonance Ionization Spectroscopy (SIRIS)

**DOI:** 10.6028/jres.093.086

**Published:** 1988-06-01

**Authors:** J. E. Parks, M. T. Spaar, L. J. Moore, W. M. Fairbank, J. M. R. Hutchinson

**Affiliations:** Atom Sciences, Inc., 114 Ridgeway Center, Oak Ridge, TN 37830; Department of Physics, Colorado State University, Fort Collins, CO 80523; Center for Radiation Research, National Bureau of Standards, Gaithersburg, MD 20899

Direct, determination of trace chemical species in solids is a fundamental analytical problem. The need to measure trace and ultratrace concentrations of species in solids requires a method that is free of matrix effects and can be carried out with minimal contamination. Sputter Initiated Resonance Ionization Spectroscopy (SIRIS) is such a method, allowing a minimum of chemical pretreatment, the chief source of contamination, and providing a highly selective and sensitive measurement of essentially any element. SIRIS is an ultrasensitive analysis technique which uses an energetic ion beam to sputter a solid sample and Resonance Ionization Spectroscopy (RIS) to selectively and efficiently ionize neutral atoms of the element of interest in the atomized cloud. RIS and SIRIS have been adequately described elsewhere [1]. A primary advantage of SIRIS technology is the potential to reduce or eliminate the matrix effect in direct solids analysis by utilizing the predominant neutral population produced by primary ion bombardment. Since the origination of the patented SIRIS technology in 1981, numerous matrix types have been analyzed for a variety of elements. We have found that for metals, semiconductors, and alloys, trace elements can be quantitated interference-free over a broad range of concentrations by using a single point calibration. Complex matrices, such as biological or geological specimens, generally require internal standardization with separated isotopes.

We have reported [1] sensitivities for SIRIS as low as 2 × 10^−9^ (atom fraction) and demonstrated linearity down to the ppm level with aluminum, vanadium, boron, copper, silicon, and selenium in standard steel samples from the National Bureau of Standards. The SIRIS measurements showed good linearity with certified values, and the relative signals from the different elements were in good agreement with the standard values.

The principal results to be reported here come from using SIRIS to investigate the spatial distribution of trace elements in specially fabricated crystals or devices. Since the ion beam is pulsed (at 30 Hz and with 1 microsecond duration) during a measurement, only about an equivalent monolayer of material is sputtered away, and the analysis pertains strictly to the surface. However, use of the unpulsed beam can remove material rapidly (ion milling), and thus provide a profile of the element being measured with depth into the sample.

Figure 1 shows a SIRIS depth profile measurement of silicon-29 implanted into gallium arsenide. The SIRIS measurements were normalized to the known peak concentration of 5×l0^18^ atoms/cm^3^ and the depth scale was calibrated by measuring the sputtered crater with a Dektak profilometer. The profile is compared to LSS theory [2] and shows good agreement at depths near the peak concentration. At larger depths the agreement is poorer, the measurement showing higher concentrations than predicted by theory. This has been explained by Shepherd [3] to be the result of channeling of the Si during implantation. He was able to avoid this effect by amorphising the gallium arsenide crystalline material before implanting. Our samples were not pre-amorphised and channeling was possible. Backgrounds near 1×10^15^ atoms/cm^3^ were demonstrated.

The matrix independence feature makes SIRIS highly desirable for the analysis of layered materials where measurements are made as a function of depth and the analysis proceeds from one material through an interface into an adjoining material. A layered sample of gallium arsenide and aluminum gallium arsenide, (GaAs/AlGaAs/GaAs), grown by MBE and doped with silicon, was depth profiled with SIRIS. Figure 2 shows the makeup of the sample and a measurement of the aluminum concentration versus depth. This indicates a 150 Å depth resolution, consistent with the 10 keV argon ion beam used for sputtering. The depth scale was determined with the profilometer and the aluminum measurement shows that the position of the aluminum layer is in agreement with the stated composition. Figure 3 shows the depth profile of the silicon dopant in this sample. The silicon concentration in the top layer of GaAs was determined electrically, based on the sum of the N and P type carriers, to be 2×10^18^ atoms/cm^3^. The electrical concentration is known to be different from the physical concentration, but the ratio of the physical concentration in the GaAs and AlGaAs layers was known from the MBE parameters to be 0.77. The ratio of the silicon concentrations in the AlGaAs layer to that in the GaAs layer was found to be 0.77 which was fortuitous since the SIRIS measurements have a precision of about 10%. The profile indicates that the silicon concentration changes at a depth consistent with the position of the interface indicated in the stated composition, and no discontinuities in the signal were observed at the interface.

The data of table 1 illustrate a different kind of analytical problem in a solid sample, the determination of a trace constituent, not in a simple, macroscopically homogeneous sample, but in a complex, heterogeneous mess, an unseparated soil or ore sample. Since admixture with graphite was used to reduce sample charging in the soil/ore samples, one set of measurements was made on graphite itself. It is clear that the absolute signal from (one isotope of) the uranium cannot be used to quantify the composition; the signal varies by about a hundred-fold over the three types of samples. However, an internal standard can be used satisfactorily, as evidenced by the nearly constant relation between the as-prepared and measured isotope ratios.

## Figures and Tables

**Figure 1:**
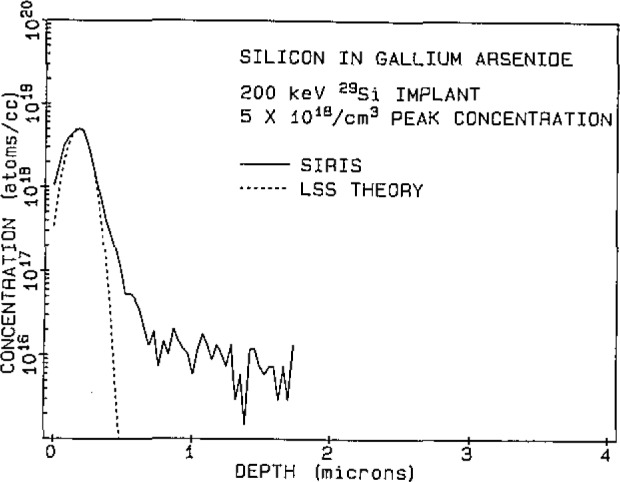
SIRIS depth profile of 200 keV silicon-29 ions implanted into gallium arsenide and compared with LSS theory.

**Figure 2:**
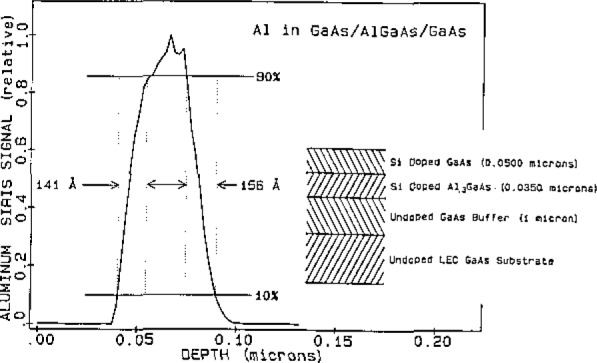
Composition of silicon doped, layered sample of GaAs/AlGaAs/GaAs and the aluminum concentration profile measured with SIRIS.

**Figure 3:**
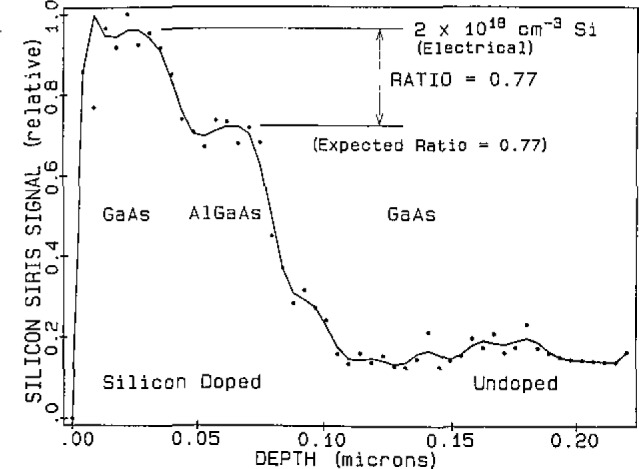
SIRIS silicon concentration profile in layered sample of GaAs/AlGaAs/GaAs.

**Table 1:** Results of isotope dilution experiments with samples of Rocky Flats soil, phosphate ore, and graphite. Samples were spiked with isotopes of U-235 and U-238 in the forms of both nitrates and oxides

Composition	Molecular form of U-235 spike	Molecular form of U-238 spike	Normalized SIRIS response	Normalized isotope ratio response
100% graphite	nitrate	nitrate	9.31	0.99
Soil with 15.2% graphite	nitrate	nitrate	3.43	1.03
100% graphite	nitrate	oxide	1.92	1.15
Soil with 19.2% graphite	nitrate	oxide	0.64	1.04
100% graphite	oxide	oxide	1.44	1.11
Soil with 23.1% graphite	oxide	oxide	0.67	1.16
Ore with 39.7% graphite	nitrate	ore	0.16	0.95
Ore with 74.8% graphite	nitrate	ore	1.52	0.72
